# Therapeutic effects of higenamine combined with [6]‐gingerol on chronic heart failure induced by doxorubicin via ameliorating mitochondrial function

**DOI:** 10.1111/jcmm.15041

**Published:** 2020-02-19

**Authors:** Jianxia Wen, Lu Zhang, Jian Wang, Jiabo Wang, Lifu Wang, Ruilin Wang, Ruisheng Li, Honghong Liu, Shizhang Wei, Haotian Li, Wenjun Zou, Yanling Zhao

**Affiliations:** ^1^ College of Pharmacy Chengdu University of Traditional Chinese Medicine Chengdu China; ^2^ Department of Pharmacy The Fifth Medical Center of PLA General Hospital Beijing China; ^3^ College of Pharmacy Zhejiang Chinese Medical University Hangzhou China; ^4^ Integrative Medical Center The Fifth Medical Center of PLA General Hospital Beijing China; ^5^ Department of Traditional Chinese Medicine The Fifth Medical Center of PLA General Hospital Beijing China; ^6^ Research Center for Clinical and Translational Medicine The Fifth Medical Center of PLA General Hospital Beijing China

**Keywords:** [6]‐gingerol, Aconiti Lateralis Radix Praeparata, chronic heart failure, doxorubicin, energy metabolism, higenamine

## Abstract

Higenamine (HG) is a natural benzylisoquinoline alkaloid isolated from *Aconitum* with positive inotropic and chronotropic effects. This study aimed to investigate the possible cardioprotective effects of HG combined with [6]‐gingerol (HG/[6]‐GR) against DOX‐induced chronic heart failure (CHF) by comprehensive approaches. DOX‐induced cardiotoxicity model in rats and H9c2 cells was established. Therapeutic effects of HG/[6]‐GR on haemodynamics, serum indices and histopathology of cardiac tissue were analysed. Cell mitochondrial energy phenotype and cell mitochondrial fuel flex were measured by a Seahorse XFp analyser. Moreover, UHPLC‐Q‐TOF/MS was performed to explore the potential metabolites affecting the therapeutic effects and pathological process of CHF. To further investigate the potential mechanism of HG/[6]‐GR, mRNA and protein expression levels of RAAS and LKB1/AMPK/Sirt1‐related pathways were detected. The present data demonstrated that the therapeutic effects of HG/[6]‐GR combination on CHF were presented in ameliorating heart function, down‐regulation serum indices and alleviating histological damage of heart tissue. Besides, HG/[6]‐GR has an effect on increasing cell viability of H9c2 cells, ameliorating DOX‐induced mitochondrial dysfunction and elevating mitochondrial OCR and ECAR value. Metabolomics analyses showed that the therapeutic effect of HG/[6]‐GR combination is mainly associated with the regulation of fatty acid metabolites and energy metabolism pathways. Furthermore, HG/[6]‐GR has an effect on down‐regulating RAAS pathway‐related molecules and up‐regulating LKB1/AMPKα/Sirt1‐related pathway. The present work demonstrates that HG/[6]‐GR prevented DOX‐induced cardiotoxicity via the cardiotonic effect and promoting myocardial energy metabolism through the LKB1/AMPKα/Sirt1 signalling pathway, which promotes mitochondrial energy metabolism and protects against CHF.

## 1 INTRODUCTION

Chronic heart failure (CHF) is the end stage of various cardiovascular diseases (CVDs). It is often accompanied by changes in the utilization of energy metabolic substrates. Also, abnormal mitochondrial function and dysfunction of energy metabolism of cardiac myocytes could lead to the progress of myocardial systolic dysfunction and left ventricular remodelling.^1^ In the advanced stages of heart failure (HF), irreversible changes often occur due to the energy metabolism disorder of cardiac myocytes.^2^ At this time, due to the reduction in mitochondrial biosynthesis and the change in mitochondrial function, mitochondrial energy metabolism is decreased resulting in the reduced ATP production, and cardiac myocytes are in the state of “energy starvation.” Recent years, the relationship between HF and myocardial energy metabolism has become a hot topic in clinical research. Drugs which can enhance myocardial mitochondrial energy metabolism may have the potential effect to treat CHF.^3^

Traditionally, the compatibility of Aconiti Lateralis Radix Praeparata (ALRP, *Fuzi* in Chinese) and Zingiberis Rhizoma (ZR, *Ganjiang* in Chinese) is a common pair of medicines in the treatment of HF, coronary heart disease (CAD), myocardial infarction and other CVDs. Studies have shown that ALRP has cardiotonic and anti‐HF effects. The water‐soluble components of ALRP, including higenamine (HG), salsolinol, coryneine chloride, fuzinoside and uracil, have been reported to be cardio cardiotonic tonic activities.^4^ ZR has a protective effect on the hypoxic and hypoglycaemic injury of cardiac myocytes. It can inhibit platelet aggregation and improve ventricular systolic and diastolic function. Simultaneously, ZR can also reduce peripheral resistance, which has a protective effect on the formation of acute heart failure (AHF) model.^5^ According to the theory of traditional Chinese medicine (TCM), both ALRP and ZR are pungent in taste and hot in nature. As a common clinical medicine pair from ancient times to nowadays, they are one of the most typical representatives reflecting the very essence of the theory of Chinese material medical compatibility. In the previous researches, the objective truth that “ALRP not exhibiting its hot property without ZR” has been proved in animals by the energy metabolism of mitochondria.^6^ However, the active components and key mechanism regulating energy metabolism remain poorly understood.

Higenamine is a plant‐based alkaloid initially isolated from the root of *Aconitum* and has been identified as the active cardiotonic component of *Aconitum*. As a traditional herb medicine, aconite has been used alone or in combination with other drugs for the treatment of HF in oriental Asia for millenaries.^7^, ^8^ Structurally, HG is similar to catecholamines, which can activate both β1‐ and β2‐adrenergic receptors (AR). β2‐AR activation explains the pharmacological effect of HG/aconite in treating CHF by enhancing the contractile response and reducing cardiomyocyte apoptosis.^9^ Also, HG is identified as a novel α1‐AR antagonist, which may contribute to its hypotensive effect and suppression platelet aggregation.^10^ Pharmaceutically, it has multiple pharmacological effects, including positive inotropic effect,^11^ dilations of blood vessels and bronchi,^12^ anti‐inflammatory activity,^13^ antispasmodics,^14^ anti‐thrombotic,^15^ anti‐oxidative^16^ and anti‐apoptotic properties.^17^ Relevant pharmacological effects and mechanisms have been reported in detail. Previous studies have shown that HG has great value on treating coronary artery disease (CVD), bradyarrhythmia, heart failure, arthritis,^11^ cerebral ischaemia‐reperfusion injury^18^ and bronchoconstriction diseases.^19^ Functional studies of isolated cardiomyocytes demonstrated its effectiveness in enhancing a contractile response.^9^ Positive inotropic drugs have been used for many years to treat patients with AHF accompanied by reduced ejection.^20^ Of note, based on the potential positive inotropic, dilating vascular and chronotropic effects, HG is an effective vasodilator in haemodynamics. It can reduce aortic impedance and systemic vascular resistance, thereby reducing afterload. These effects are similar to those of isoproterenol (ISO). Recent years, HG was approved by the China Food and Drug Administration for the clinical study. Currently, the phase III clinical trial of HG has been completed and achieved good results.^21^ These properties suggest that HF could be potentially treated using HG with its inotropic and chronotropic effects. Studies specifically designed to assess the potential role and mechanism of HG in the treatment of HF are necessary.

Ginger has been widely used as traditional medicine for thousands of years. 6‐gingerol ([6]‐GR), a major bioactive constituent of ginger, is the most abundant and pungent gingerol in ginger. It has many effective pharmacological effects, such as cardiotonic, anti‐angiogenesis, anti‐inflammatory, anti‐cancer, antipyretic and anti‐ageing.^22^ Notably, [6]‐GR is a novel angiotensin II receptor (AT1) antagonist and offers some beneficial effects on cardiovascular diseases.^23^ Chen et al investigated the active components of Sini decoction (a classic prescription of TCM) and the corresponding potential cardioprotective mechanisms. The results indicated that the combined use of HG and [6]‐GR shows cardioprotective function against DOX‐induced cardiotoxicity via activating the PI3K/Akt signalling pathway.^16^ Moreover, our previous study has shown that HG combined with [6]‐GR (HG/[6]‐GR) can protect H9c2 cardiomyocyte from doxorubicin (DOX)‐induced cardiomyocyte toxicity, which alleviates cell mitochondrial respiratory impairment and energy metabolism disorder.^24^ HG and [6]‐GR may serve as the active components of Aconiti Lateralis Radix Praeparata (ALRP, *Fuzi* in Chinese) and Zingiberis Rhizoma (ZR, *Ganjiang* in Chinese), respectively. However, previous studies only clarified the possibility of HG/[6]‐GR in protecting H9c2 cells from DOX‐induced cardiotoxicity in vitro. It has not been studied whether HG/[6]‐GR has the therapeutic effect on CHF in a holistic animal model.

In the current study, conventional pharmacology, molecular biology, seahorse XFp, metabolomics and other multidisciplinary technologies have been applied to investigate whether HG in combination with [6]‐GR could therapy DOX‐induced CHF in rats and protect H9c2 cardiomyocytes from DOX‐induced mitochondrial function impairment as well as the underlying molecular mechanisms in vivo and in vitro. Results suggested that HG/[6]‐GR indeed has therapeutic efficacy on CHF via improving myocardial haemodynamics and mitochondrial energy metabolism. The results indicate that the molecular mechanisms underlying the cardio‐protective and therapy effects of HG/[6]‐GR may be related to contractility in rat cardiomyocytes and improvement on mitochondrial energy metabolism.

## 2 MATERIALS AND METHODS

### 2.1 Materials

Standards of HG (Cat No. CHB180121), [6]‐GR (Cat No. CHB180306) and DOX (Cat No. CHB180204) were purchased from Chroma Biotechnology Co. Ltd. Doxorubicin hydrochloride for injection was purchased from Shenzhen Main Luck pharmaceutical Inc (Batch number: 1809E2). Heparin sodium injection was purchased from Changzhou Qianhong Bio‐pharma Co. Ltd (Batch number: 151805061A). Dobutamine hydrochloride (DH) injection (Batch number: 1803203) was obtained from SPH NO.1 Biochemical & Pharmaceutical CO., LTD.

### 2.2 Animal care

All animal procedures have complied with the Guiding Principles for the Care and Use of Laboratory Animals of China and Institutional Animal Care and Use Committee of the Fifth Medical Center of PLA General Hospital. All animal studies were approved by the Committee on the Ethics of Animal Experiments of the Fifth Medical Center of PLA General Hospital (Approval ID: IACUC‐2018‐010).

### 2.3 Animal handling

Male SD rats weighing 200 ± 20 g (Permission No. SCXK‐(jing) 2018‐0010) were provided by the Beijing Keyu Animal Breeding Center (Beijing, China), and the quality control department was National Institutes for Food and Drug Control. CHF model was established by DOX hydrochloride injection (2.5 mg/kg, accumulative 15 mg/kg) and evaluated by an RM6240 series multichannel physiological signal acquisition as our previous study.^25^ Then, CHF model rats were randomly divided into five groups: (A) DOX group; (B) DH‐positive group (50 μg/kg/d), (C) HG group (5 mg/kg/d), (D) [6]‐GR group (5 mg/kg/d) and (E) HG/[6]‐GR compatibility group (10 mg/kg/d). Rats were intraperitoneally injected with the corresponding drugs once a day for 7 days. HG and [6]‐GR in 5 mg/kg/d showed superior efficacy in our previous study. Heart function was detected after the final injection. Serum samples and heart tissue were collected and stored at −80°C for the following detection.

### 2.4 Detection of pharmacodynamic indicators

Serum levels of renin (RE), angiotensin II (Ang‐II), aldosterone (ALD) and endothelin 1 (ET‐1) were detected by a Synergy hybrid reader (BioTek). Left ventricular myocardial tissue was cut longitudinally and fixed with 4% paraformaldehyde. Haematoxylin‐eosin (H&E) staining was performed for detecting myocardial morphological changes. Paraffin‐embedded sections were observed under a Nikon microscope and by a Pro‐Plus 7200 software.

### 2.5 Cell culture and cell viability assay

The H9c2 rat cardiomyocyte cell lines were obtained from the Cell Resource Centre (IBMS, CAMS/PUMC). Cells were cultured with Dulbecco's modified Eagle's medium (DMEM) containing 10% foetal bovine serum (FBS), supplemented with 100 × penicillin‐streptomycin (Cat No. CC004, MACGENE). H9c2 cells were cultured in a CO_2_ constant‐temperature incubator (37°C, saturated humidity atmosphere of 95% air and 5% CO_2_). H9c2 rat cardiomyocyte cells were pre‐treated with 20 μmol/L HG, 20 μmol/L [6]‐GR or 20 μmol/L HG plus 20 μmol/L [6]‐GR following 5 μmol/L DOX for 24 hours as our previous study.^24^

### 2.6 Cell metabolomics

#### 2.6.1 Extraction of intracellular metabolites

Cell samples were collected and washed three times with PBS. 1 mL of methanol‐water (4:1, V/V solution, standing 20 minutes at 4°C before use) was successively added to each sample, which was sealed and put into liquid nitrogen for crushing and quenching. The supernatant of the sample was centrifuged at 4°C, 16 000 *g* for 10 minutes to precipitate the protein. Then, the supernatant was transferred to the autosampler vials through 0.22‐μm microporous membrane for metabolomics research. The quality control (QC) sample was prepared by mixing 10 μL aliquots of all individual H9c2 cell samples to evaluate the reproducibility and stability of pre‐treated cells.

#### 2.6.2 UHPLC‐Q‐TOF/MS analysis

In this study, an Agilent 1290 series UHPLC system (Agilent Technologies, United States) was used to analyse the intracellular metabolic profile of H9c2 cardiomyocytes. A ZORBAX RRHD 300 SB‐C18 column (100 mm × 2.1 mm, 1.8 μm, Agilent Technologies) was applied for chromatographic separation. Mobile phase A was 0.1% formic acid in acetonitrile, and phase B was 0.1% formic acid in water. The column temperature was kept at 30°C, and the flow rate was 0.30 mL/min. The gradient elution of mobile phase A was set as follows: 0‐1.0 minutes, 95%; 1.0‐9.0 minutes, 95%‐60%; 9.0‐19.0 minutes, 60%‐10%; 19.0‐21.0 minutes, 10%‐0%; 21.0‐25.0 minutes, 0%. All samples were preserved at 4°C with a 4 μL injection volume during the analysis.

Then, mass spectrometry was carried out on an Agilent 6550A Q‐TOF/MS (Agilent Technologies, United States) with electrospray ionization (ESI) mode. The mass range was set from 80 to 1000 m/z, and the gas flow was set as 11 L/min. The conditions in positive ionization mode were set as follows: electrospray capillary voltage, 4.0 kV; gas temperature, 225°C; nebulizer, 45 pisg. And conditions in negative ionization mode were set as follows: electrospray capillary voltage, 3.0 kV; gas temperature, 200°C; nebulizer, 35 pisg. The other parameters were as follows: sheath gas temperature, 350°C; sheath gas flow rate, 12 L/min; nozzle voltage, 500 V.

#### 2.6.3 Data processing and multivariate statistical analysis

Statistical analysis in MetaboAnalyst 4.0^26^ online system was used to normalize the original data. The exported “data_normalized.usp” file was then imported into SIMCA‐P 14.1 software (Umetrics, Umea, Sweden) for further multivariate statistics, including the principal component analysis (PCA) and partial least squares‐discriminant analysis (PLS‐DA). The metabolites with a higher significant difference in projection (VIP > 1) and correlation coefficient value (|Pcorr| > 0.58) in the OPLS‐DA model were analysed for sample *t* test. To avoid the over‐fitting of the PLS‐DA model, 100 iteration permutation tests were performed. Next, a MassHunter Profinder software (version B.06.00, Agilent) was used to extract and analyse the mass spectra data. An identified retention time and m/z of every peak would be presented. All potential metabolites were preliminarily identified by using the online biochemical database METLIN (http://metlin.scripps.edu/) and HMDB database (http://www.hmdb.ca/). MetaboAnalyst 4.0 was used for the pathway enrichment analysis of the previously identified potential metabolites. In this study, potential biomarkers and possible mechanisms of HG and [6]‐GR in the treatment of DOX‐induced H9c2 cardiomyocyte injury were elucidated by cell metabolomics strategy.

### 2.7 Analysis of cell energy phenotype and cell extracellular flux

Seahorse XFp Cell Energy Phenotype Test and Extracellular Flux (XFp) analyser (XFp, Seahorse Biosciences, MA) were performed according to the provided protocol. Cell processing was performed as our previous studies.^24^ After subsequent mixing and injection of the compounds (10 μmol/L oligomycin and 10 μmol/L carbonyl cyanide‐4‐(trifluoromethoxy) phenylhydrazone [FCCP]), the values of oxygen consumption rate (OCR) and extracellular acidification rate (ECAR), stressed OCR and stressed ECAR were determined in all wells three times. As cardiomyocyte oxidative function is mostly derived from fatty acid metabolism, several compounds (4 μmol/L etomoxir, 3 μmol/L BPTES and 2 μmol/L UK5099) were subsequently injected and mixed to evaluate the dependency, capacity and flexibility of H9c2 cells in oxidizing mitochondrial fuels and fatty acids. Seahorse XFp metabolic data were normalized by cell counting (6 × 10^3^ cells/well). Besides, data were analysed using the Seahorse XF test report analysis.

### 2.8 RNA extraction and RT‐PCR analysis

Total RNA was extracted from approximately 80 mg of heart tissue using TRIzol reagent (BioFlux). RNA was transformed into cDNA by a reverse transcription kit (Promega, Madison, USA). RT‐PCR was performed on a QuantStudio™ Real‐Time PCR System version 1.3 (Applied Biosystems by Thermo Fisher Scientific). During the experiment, all the steps from sample extraction and storage to RNA extraction, testing the RNA samples, reverse transcription, primer design and validation and so on were strictly performed according to the operation prompt of Minimum Information for the Publication of Quantitative Real‐Time PCR Experiments (MIQE).^24^ The related primer sequences are shown in Table S1. For the data analysis, the relative mRNA expression levels of these genes were calculated based on 2^−ΔΔCt^ calculations to the β‐actin endogenous reference.

### 2.9 Immunohistochemical staining

Immunohistochemistry staining was performed as follows. The cardiac tissue was fixed with 4% paraformaldehyde and embedded in paraffin. ACE Rabbit Polyclonal antibody (Catalog Number: 24743‐1‐AP, Proteintech, 1:50), AGTR1 Rabbit Polyclonal antibody (Catalog Number: 25343‐1‐AP, Proteintech, 1:20) and NRG1 Mouse Monoclonal antibody (Catalog Number: 66492‐1‐Ig, Proteintech, 1:50) were used. The images were photographed on NIS Elements Imaging Software version 4.0 at 200 magnification (Nikon).

### 2.10 Protein isolation and Western blotting

Total protein was isolated from approximately 80 mg of heart tissue with RIPA buffer (high) (Lot. No. 20190510, Solarbio) containing 1/100 PMSF (Lot. No. 20190103, Solarbio). BCA protein assay kit (Lot. No. 20190524, Solarbio) was used to detect protein concentration. The following primary antibodies LKB1 (D60C5) Rabbit mAb (dilution: 1:1,000, Cell Signaling Technology), Anti‐AMPK alpha 1 antibody [Y365] ab32047 (dilution: 1:3,000, Abcam), Anti‐SIRT1 antibody [19A7AB4] ab110304 (dilution: 1:1,000, Abcam), Anti‐PGC1 alpha antibody ab54481 (dilution: 1:1,000, Abcam), Mouse anti‐β‐Actin (dilution: 1:2,000, Boster Biological Technology) were used. Images were acquired by using a Western blotting detection system (Quantity One, Bio‐Rad Laboratories, USA).

### 2.11 Statistical analysis

Statistical analysis was performed using SPSS 23.0 software program (Chicago, United States) and GraphPad Prism 8.2.0 software (GraphPad Software). All data were presented as the mean ± standard deviation (SD). The differences between the two groups or multiple groups were calculated by a *t* test and one‐way analysis of variance (ANOVA). The difference was considered statistically significant at *P* < .05 and highly significant at *P* < .01.

## 3 RESULTS

### 3.1 Effects of HG/[6]‐GR on cardiac function in rats

**Figure 1:**
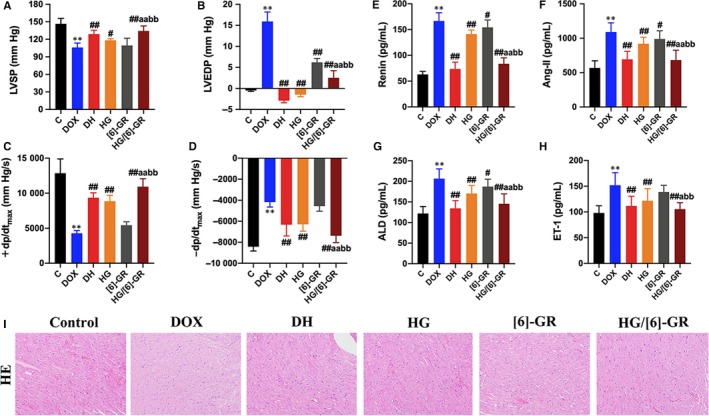
HG combined with [6]‐GR ameliorates the myocardial function in rats. Haemodynamic parameters, including (A) LVSP; (B) LVEDP; (C) +dp/dtmax; (D) −dp/dtmax, were measured by multichannel physiological signal acquisition system. HG/[6]‐GR reduces serum Renin (E), Ang‐II (F), ALD (G) and ET‐1 (H) levels in CHF rats. (I) Representative photomicrographs for HE staining of heart histological changes. **P < .01, compared with the control group. #*P* < .05 and ##*P* < .01, compared with the DOX group. a*P* < .05, aa*P* < .01, compared with the HG group. bb*P* < .01 compared with the [6]‐GR group. (HE stained, 200× magnification)

The results in Table S2 showed that LVSP and +dp/dt_max_ decreased significantly in the DOX group, while LVEDP and −dp/dt_max_ increased substantially in the DOX group. The +dp/dt_max_ reduced to 50% of the control group indicated that the CHF rat model had been successfully established. The impact of HG/[6]‐GR on cardiac function was assessed by detecting the haemodynamic parameter levels of the heart. Compared with the control group, the haemodynamic parameter levels of LVSP and +dp/dt_max_ in the DOX group were substantially decreased (*P* < .01). Conversely, the levels of LVSP and +dp/dt_max_ in the DH, HG and HG/[6]‐GR groups increased significantly but LVEDP and −dp/dt_max_ decreased markedly compared with the DOX group. However, [6]‐GR could only significantly reduce the level of LVEDP. Notably, the HG/[6]‐GR group had a better effect on improving cardiac function than HG and [6]‐GR used alone (Figure 1A‐D).

### 3.2 Serum levels of renin, Ang‐II, ALD and ET‐1 in various groups

As shown in Figure 1E‐H, compared with the control group, serum levels of renin, Ang‐II, ALD and ET‐1 in the DOX group were markedly increased (*P < *0.01). Compared with the DOX group, these indices were significantly reduced when rats were treated with DH and HG/[6]‐GR (*P < *0.01). Notably, these serum biochemical indicators had been significantly reduced by treated HG alone. Furthermore, the HG/[6]‐GR group was almost equal to the DH group. Hence, [6]‐GR may enhance the role of HG in the treatment of CHF, for which the HG/[6]‐GR couple has better therapeutic efficacy than their single use.

### 3.3 Histological examination of heart damage

Histological examination of heart damage in Figure 1I showed that the connective tissue structure of myocardium in the control group was clear with normal chromatin. The myofibrils of myocardial tissue are arranged neatly with the same size and shape of nuclei. There was no hypertrophy, necrosis, atrophy, rupture and interstitial cell proliferation in the control group. On the contrary, the myocardium showed focal necrosis accompanied by myocardial fibrosis. Myocardial interstitial oedema and infiltration of inflammatory cells were observed. Myocardial fibres in the DH and HG/[6]‐GR groups were locally ruptured and disorderly arranged, but atrophic degeneration and interstitial fibrous tissue proliferation were relatively small. Necrotic myocardial cells were significantly reduced. The HG and [6]‐GR groups had different degrees of myocardial disorders. However, myocardial fibrosis and inflammatory cell infiltration were not obvious (Figure 1I).

### 3.4 Evaluation of the metabolic disturbances in DOX‐induced H9c2 cardiomyocyte injury

**Figure 2:**
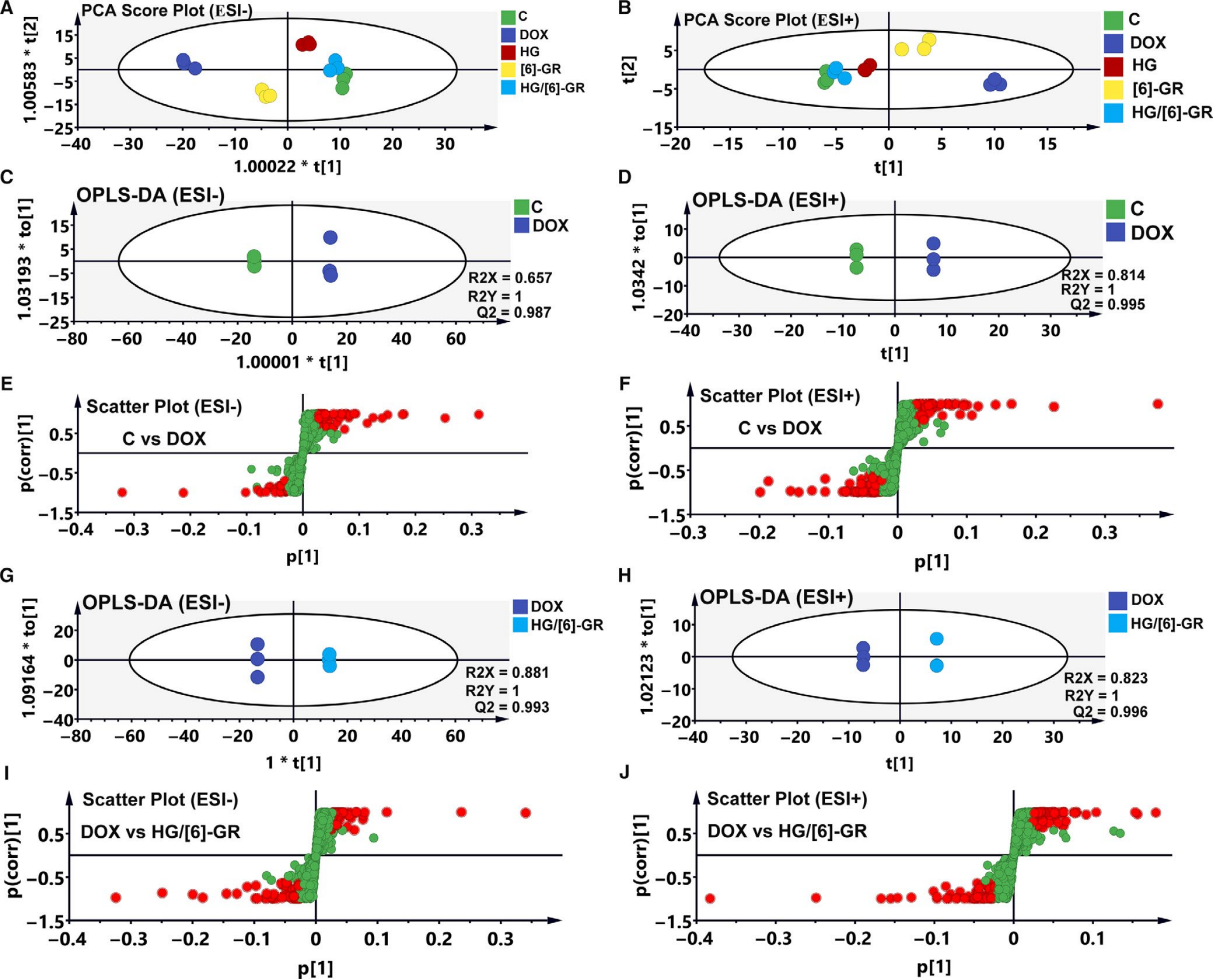
Metabolic profiles and differentiation of the control, DOX and HG/[6]‐GR groups by multivariate analysis. The score scatter plots of the control, DOX and HG/[6]‐GR‐treated groups from PCA data in the ESI− mode (A) and ESI+ mode (B); OPLS‐DA score scatter plots for the pairwise comparisons between the control and DOX groups in the ESI− mode (C) and ESI+ mode (D); S‐plot of the OPLS‐DA model for the control and DOX groups in the ESI− mode (E) and ESI+ mode (F); OPLS‐DA score scatter plots for the pairwise comparisons between the DOX and HG/[6]‐GR‐treated groups in the ESI− mode (G) and ESI+ mode (H); S‐plot of the OPLS‐DA model for the DOX and HG/[6]‐GR groups in the ESI− mode (I) and ESI+ mode (J)

**Figure 3:**
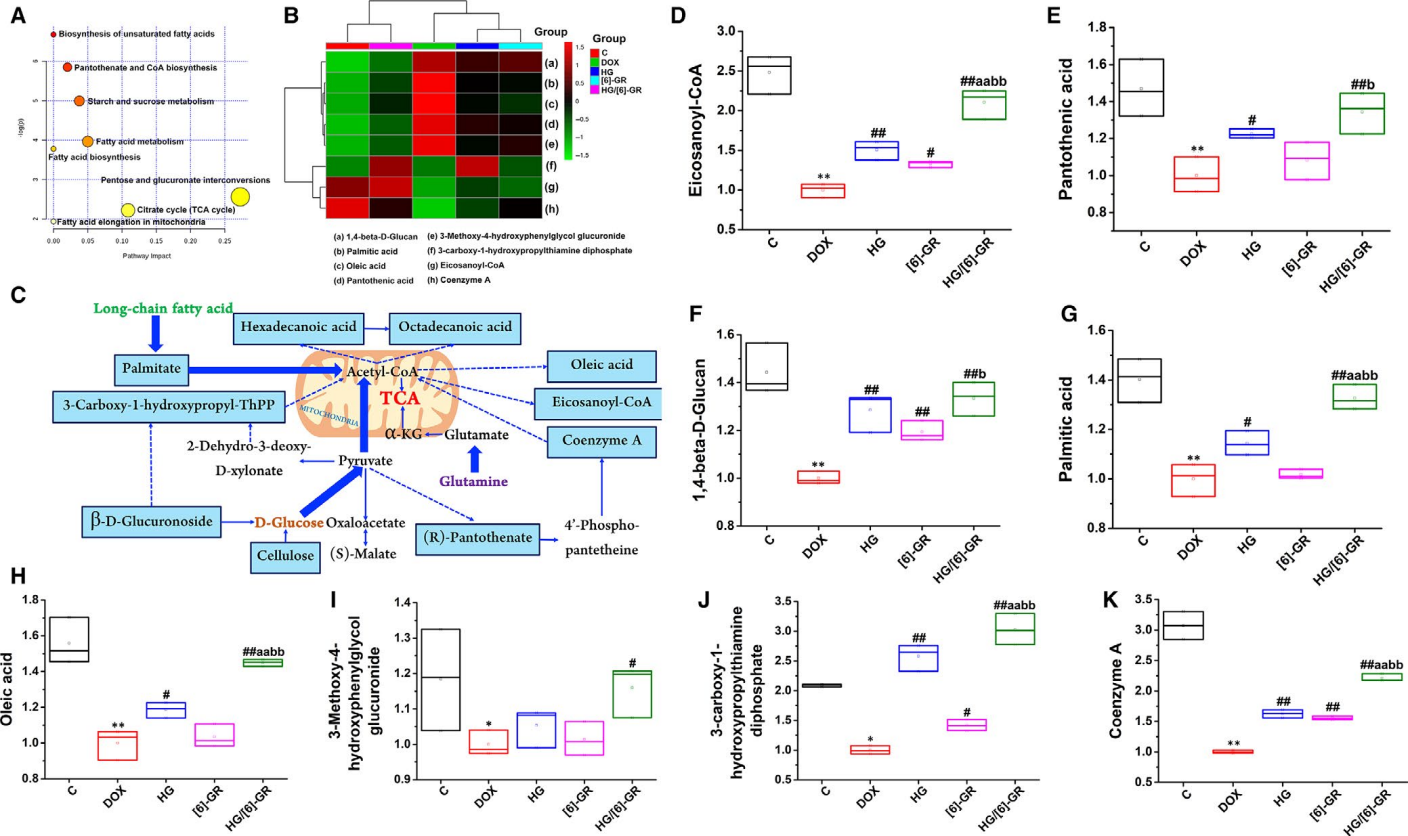
Potential metabolites changes and related metabolomic pathway involved in the treatment of HG/[6]‐GR on DOX‐induced CHF. (A) The metabolomic pathway involved in the effects of HG/[6]‐GR on DOX‐induced CHF. (B) The cluster heatmap of metabolites both in each group. (C) Signalling networks associated with the differentially expressed metabolic pathways. Potential metabolites changes in DOX‐induced CHF with HG/[6]‐GR treatment in Eicosanoyl‐CoA (D); Pantothenic acid (E); 1,4‐beta‐D‐Glucan (F); Palmitic acid (G); Oleic acid (H); 3‐Methoxy‐4‐hydroxyphenylglycol glucuronide (I); 3‐carboxy‐1‐hydroxypropylthiamine diphosphate (J); Coenzyme A (K). **P < .01, compared with the control group; #*P* < .05, ##*P* < .01, compared with the DOX group. aa*P* < .01 compared with the HG group. b*P* < .05 and bb*P* < .01 compared with the [6]‐GR group

As metabolomics could comprehensively evaluate the metabolites in cells, the pattern recognition approaches such as “unsupervised” PCA and “supervised” PLS‐DA were performed to assess the metabolic disturbances in DOX‐induced H9c2 cardiomyocyte injury. The clustered scatter in the control, DOX, HG, [6]‐GR and HG/[6]‐GR groups indicated the similar metabolomic compositions, while the dispersed scatter displayed in different groups. A cluster heatmap (Figure 3B) and PCA data were analysed to evaluate the distributions and the differences between the groups either in negative mode or in positive mode (Figure 2A,B, respectively). An obvious separation was observed between the control and DOX groups in both negative mode and positive mode, indicating that the metabolic profiles had altered owing to intraperitoneal injection of DOX. The groups pre‐treated with HG, [6]‐GR, and HG/[6]‐GR moved towards the control group compared with the DOX group, illustrating that HG, [6]‐GR and HG/[6]‐GR effectively regulated metabolic parameters. Particularly, the HG/[6]‐GR group was much closer to the control group than the others, which indicated a potent therapeutic effect of CHF. However, the [6]‐GR group was relatively close to the DOX group in ESI− mode and ESI+ mode, which suggested that [6]‐GR might have a weaker effect on regulating metabolic disorders induced by DOX. In particular, the therapeutic effect observed for the HG/[6]‐GR was much better than that observed in the HG and [6]‐GR used alone.

As HG/[6]‐GR had the best therapeutic efficacy on DOX‐induced CHF in this study. OPLS‐DA was used to investigate the differences between the control group and the DOX group, the DOX group and the HG/[6]‐GR group to find potential biomarkers for the treatment of CHF. The left side of Figure 2 displays the results of ESI‐ mode and the right side for the ESI+ mode in the OPLS‐DA model. As shown in Figure 2C,D, the OPLS‐DA model was constructed based on the control and DOX data. The DOX group could be separated from the control group very clearly. The model demonstrated good predictive ability with an R2Y (cum) of 1 and a Q2 (cum) of 0.987 in ESI‐ mode and R2Y (cum) of 1 and a Q2 (cum) of 0.995 in ESI+ mode. Similarly, the OPLS‐DA model was constructed based on the DOX and HG/[6]‐GR data (Figure 2G,H). The DOX group could be separated from the HG/[6]‐GR group clearly. The R2Y (cum) and Q2Y (cum) were 1 and 0.993 in ESI‐ mode, 1 and 0.996 in ESI+ mode, respectively. Data analysis between the DOX and HG group, DOX and [6]‐GR group in these two modes was also conducted and was shown in Figure S1. Figure 2E,F,I,J showed the S‐plots of the control and DOX group, DOX and HG/[6]‐GR group. The same process was performed for the data from the DOX and HG group, DOX and [6]‐GR group (Figure S1C,D and Figure S1I,J).

### 3.5 Identification of potential biomarkers in CHF treatment and correlation analysis with energy metabolism indicators

Next, potential biomarkers for CHF treatment were identified. According to the thresholds of VIP values ≥ 1.0 and |p(corr)| values ≥ 0.58, the variables that substantially contributed to the clustering and identification were identified. All thresholds were obtained after OPLS‐DA processing. Then, candidate variables were selected from the control group, DOX group and HG/[6]‐GR group for the folding change and variance analysis. Candidates with significant differences between groups and folding changes more than twice were identified as candidate biomarkers for METLIN and MetaboAnalyst. Finally, eight potential biomarkers were selected as biomarkers for the treatment of CHF, and their compound name, formula, mass (m/z), retention time and variation trends were summarized in Table 1 for the further understanding of therapeutic efficacy. The potential mechanism of HG/[6]‐GR on CHF and the changes in eight possible metabolites were assessed. Compared with the control group, DOX significantly reduced peak area of Eicosanoyl‐CoA, Pantothenic acid, 1,4‐beta‐D‐Glucan, Palmitic acid, Oleic acid, 3‐Methoxy‐4‐hydroxyphenylglycol glucuronide, 3‐carboxy‐1‐hydroxypropylthiamine diphosphate and Coenzyme A. However, HG/[6]‐GR has a change effect in these metabolites, indicating the formation of substrates in the mitochondrial energy metabolism (Figure 3C). In addition, a clustered heatmap and PCA based on the potential biomarkers data were constructed to determine the distributions and find the differences between the groups (Figure 3B). Taken together, the results demonstrated that the HG/[6]‐GR group showed significant therapeutic effects on CHF. In particular, the therapeutic effect observed for the HG/[6]‐GR was much better than that observed in the HG and [6]‐GR groups (Figure 3D‐K).

**Table 1:** Identified metabolites of the H9c2 cells from different groups

No	Compound name	Formula	Mass (m/z)	RT (min)	Ratio changes (significance)	
					Control/DOX	HG/[6]‐GR/DOX
1	Eicosanoyl‐CoA	C_41_H_74_N_7_O_17_P_3_S	1061.422	22.68	2.48**	2.10##
2	Pantothenic acid	C_9_H_17_NO_5_	219.1107	3.45	1.47**	1.34##
3	1,4‐beta‐D‐Glucan	C_18_H_32_O_18_	536.1428	18.1	1.44**	1.33##
4	Palmitic acid	C_16_H_32_O_2_	256.237	18.47	1.40**	1.33##
5	Oleic acid	C_18_H_34_O_2_	282.2566	17.25	1.56**	1.45##
6	3‐Methoxy‐4‐hydroxyphenylglycol glucuronide	C_25_H_38_O_9_	482.2389	7.41	1.18^*^	1.16^#^
7	3‐carboxy‐1‐hydroxypropylthiamine diphosphate	C_16_H_25_N_4_O_10_P_2_S	527.2989	14.03	2.09^*^	3.03##
8	Coenzyme A	C_21_H_36_N_7_O_16_P_3_S	767.1139	19.49	3.08**	2.21##

** *P* < .01 compared with the control group.

^##^ *P* < .01 compared with the DOX group.

### 3.6 Related pathways of differential metabolites

MetaboAnalyst 4.0 was used for the detailed pathway analysis, which focuses on exploratory statistical analysis, functional interpretation and advanced statistics for translational metabolomic studies. Metabolic pathway analysis indicated that eight potential metabolites were important for CHF treatment. As shown in Figure 3A and Table 2, fatty acid metabolism‐related signalling pathways, such as biosynthesis of unsaturated fatty acids, fatty acid metabolism, fatty acid biosynthesis and fatty acid elongation in mitochondria, were mainly enriched as the crucial signalling pathway in the HG/[6]‐GR‐treated CHF. Notably, biomarker enrichment analysis and metabolic analysis showed that the citrate cycle, also known as the tricarboxylic acid cycle (TCA cycle), was recognized as one of the related pathways. TCA cycle is a pivotal link among three major substances (including sugar, fat, protein and even nucleic acid metabolism) and energy metabolism. Combined with fatty acid metabolism, HG/[6]‐GR might play a therapeutic role in CHF by regulating fatty acid metabolism and mitochondrial energy metabolism‐related pathways, which is consistent with the results of fatty acid metabolism in seahorse analysis. In addition, pantothenate and CoA biosynthesis, starch and sucrose metabolism, pentose and glucuronate interconversions were also identified as the potential metabolic pathways, which were contributed to energy metabolism (Figure 3C). The matching status, *P* value, −log(p) and the impact of each metabolic pathway are listed in Table 2.

**Table 2:** Results of integrating enrichment analysis of biomarkers with MetaboAnalyst 4.0

No	Pathway name	Match status	*P*	−log(p)	Impact
1	Biosynthesis of unsaturated fatty acids	3/42	.0012623	6.6748	0
2	Pantothenate and CoA biosynthesis	2/15	.0028842	5.8485	0.02041
3	Starch and sucrose metabolism	2/23	.0067918	4.992	0.03778
4	Fatty acid metabolism	2/39	.018998	3.9634	0.05008
5	Fatty acid biosynthesis	2/43	.022886	3.7772	0
6	Fatty acid elongation in mitochondria	1/27	.14441	1.9351	0
7	Citrate cycle (TCA cycle)	1/20	.10884	2.2178	0.10893
8	Pentose and glucuronate interconversions	1/14	.077335	2.5596	0.27273

### 3.7 HG/[6]‐GR combination promotes mitochondrial respiratory function

**Figure 4:**
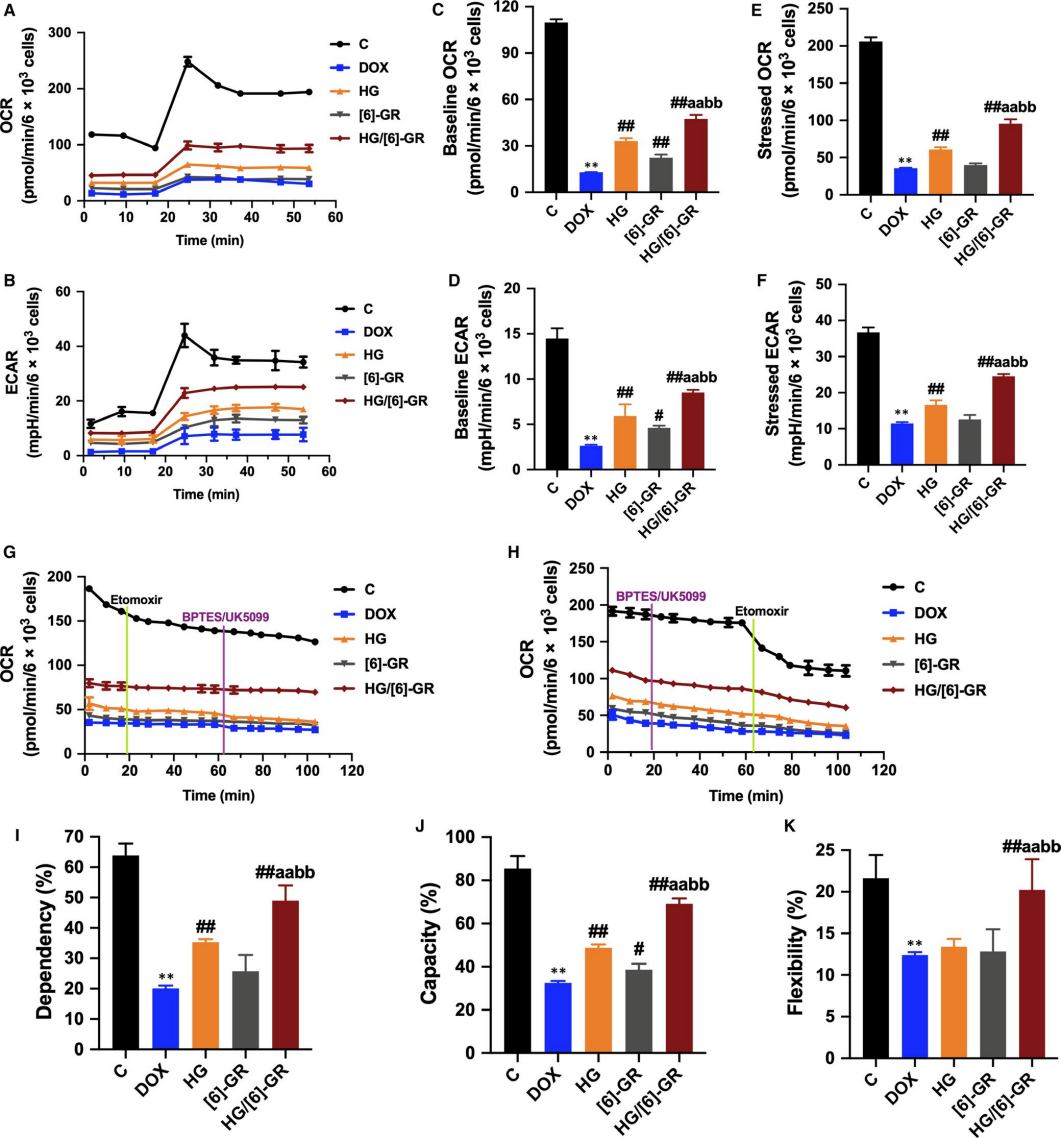
HG combined with [6]‐GR mitigates the inhibition effect of DOX on mitochondrial respiration and attenuates H9c2 cells from DOX‐induced decreases in mitochondrial fuel flexibility. Mitochondrial respiration was measured by a Seahorse XFp apparatus to detect OCR and ECAR values. During testing, H9c2 cells were treated with 10 μmol/L oligomycin and 10 μmol/L FCCP. (A) OCR. (B) ECAR. (C) Baseline OCR. (D) Baseline ECAR. (E) Stressed OCR. (F) Stressed ECAR. Mitochondrial substrate analysis was determined. During testing, H9c2 cells were treated with 4 μmol/L etomoxir and 3 μmol/L BPTES/2 μmol/L UK5099 in succession. (G) Effects of HG/[6]‐GR combination on fuel dependency in terms of the fatty acid oxidation pathway. (H) Effects of HG/[6]‐GR combination on fuel capacity in terms of the fatty acid oxidation pathway and oxidation rates of fatty acids expressed in dependency (I), capacity (J) and flexibility (K) to maintain baseline OCR levels determined with the Seahorse XFp respirometer. **P < .01, compared with the control group. #*P* < .05 and ##*P* < .01, the compared with DOX group. a*P* < .05 and aa*P* < .01 the compared with HG group. b*P* < .05 and bb*P* < .01 compared with the [6]‐GR group

To assess whether the observed toxicity of DOX is correlated with mitochondrial respiration defects, a Seahorse XFp analyser was used to determine the OCR and ECAR values of H9c2 cells. DOX specifically impaired both OCR and ECAR values in H9c2 cells (Figure 4A,B). Compared to the DOX group, baseline OCR (Figure 4C), baseline ECAR (Figure 4D), stressed OCR (Figure 4E) and stressed ECAR (Figure 4F) were significantly promoted in H9c2 cells when pre‐treated with the combination of 20 μmol/L HG and 20 μmol/L [6]‐GR (*P* < .01, *P* < .01,* P* < .01 and* P* < .01, respectively). These results demonstrated that DOX treatment causes mitochondrial respiration dysfunction and that the combined use of HG/[6]‐GR attenuates these effects.

### 3.8 HG/[6]‐GR combination promotes mitochondrial energy metabolism

In view of the crucial role of fatty acid metabolism to energy metabolism in metabolomics studies, fatty acid metabolism was blocked with etomoxir (carnitine palmitoyltransferase‐I inhibitor of fatty acid mitochondrial import), and the glucose and glutamine pathways were inhibited with UK5099 and BPTES, respectively. DOX‐treated H9c2 cells had a decreased OCR and dependency on fatty acids compared to the control groups, while the HG‐treated groups had an increased OCR and dependency to maintain baseline respiration, especially the HG and [6]‐GR combination groups (*P* < .01) (Figure 4G,I). After inhibition of the glucose and glutamine pathway followed by blockade of fatty acid metabolism, an increased capacity of fatty acid utilization was observed in H9c2 cells when other fuel pathways were inhibited (*P* < .01) (Figure 4H,J). There was a significant decrease in the flexibility of fatty acid oxidation in the DOX group. And the HG/[6]‐GR combination group showed a significant increase (*P* < .01) (Figure 4K). These results indicated that when the fatty acid pathway is inhibited, the combination use of HG and [6]‐GR resulted in increased flexibility in glucose and glutamine capacity to maintain cellular physiology.

### 3.9 Effect of HG/[6]‐GR on the mRNA expression of myocardial microvasculature

**Figure 5:**
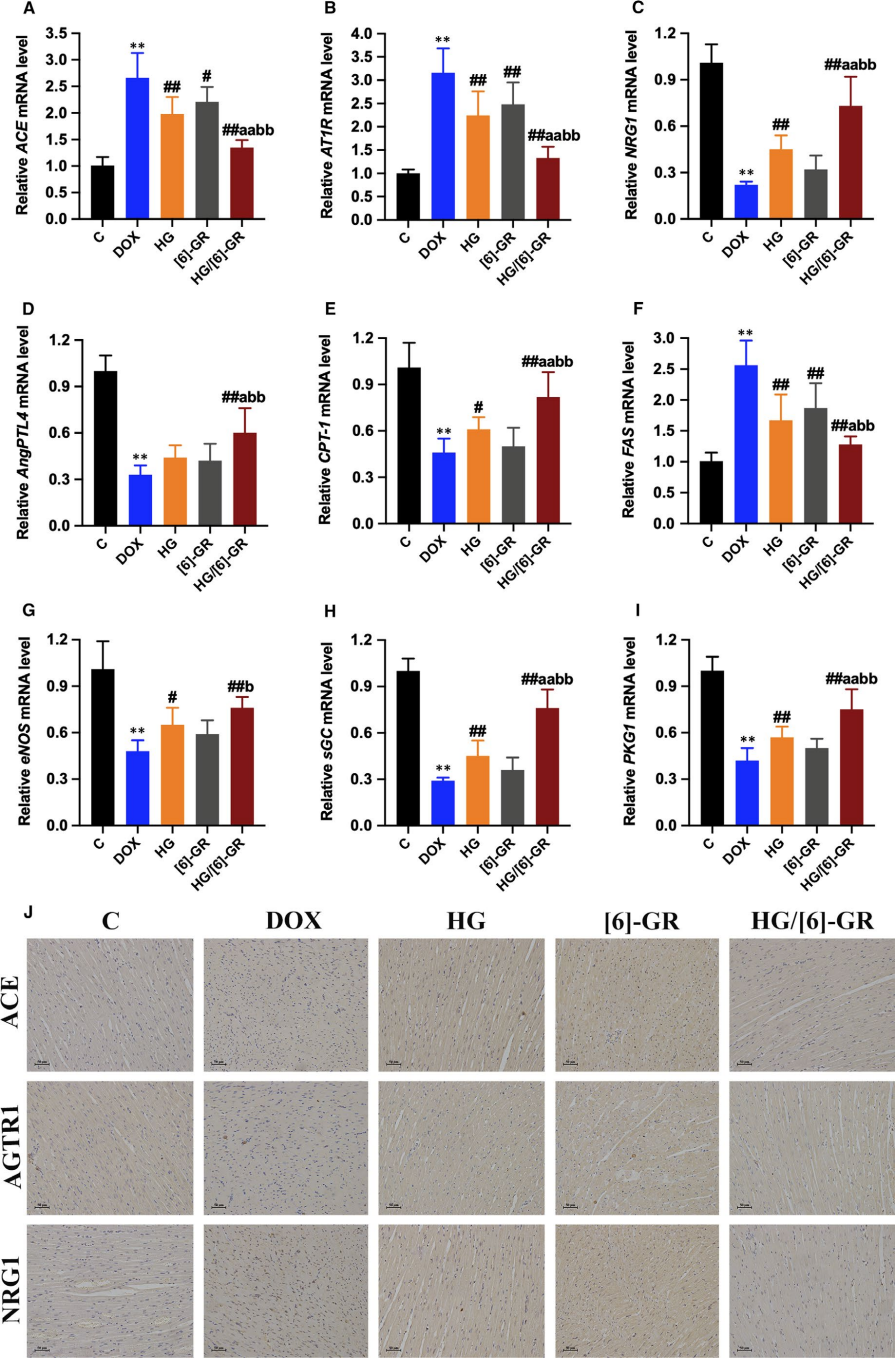
Effect of HG/[6]‐GR couple on the relative mRNA expressions in rats with CHF. The relative mRNA expression levels of ACE (A), AT1R (B), NRG1 (C), AngPTL4 (D), CPT‐1 (E), FAS (F), eNOS (G), sGC (H) and PKG1 (I) were analyzed by RT‐PCR. (J) The level of ACE, AGTR1 and NRG1 in heart tissues was measured using immunohistochemical staining. Data are expressed as mean ± SD. **P < .01, compared with the control group; #*P* < .05, ##*P* < .01, compared with the DOX group; a*P* < .05, aa*P* < .01, compared with the HG group; b*P* < .05, bb*P* < .01, compared with the [6]‐GR group. (Immunohistochemical staining, 200× magnification)

As shown in Figure 5A,B, DOX significantly increased the relative mRNA level of *ACE* and *AT1R* (*P* < .01). Conversely, the expression levels of *ACE* and *AT1R* in the myocardium of rats in the HG/[6]‐GR group were significantly decreased (*P* < .01). In addition, the results showed that the relative mRNA levels of *NRG1* and *AngPTL4* in the DOX group decreased significantly (Figure 5C,D) (*P* < .01). However, HG/[6]‐GR can significantly increase the relative mRNA levels of *NRG1* and *AngPTL4* in myocardial tissue (*P* < .01). In view of the key role of fatty acid metabolism in energy metabolism, this study further explored the effects of HG/[6]‐GR on the gene expression of *CPT‐1* and *FAS*. HG/[6]‐GR can substantially increase the *CPT‐1* level decreased by DOX (*P* < .01), and decrease the *FAS* level increased by DOX (*P* < .01), which indicated that HG/[6]‐GR could regulate fatty acid metabolism (Figure 5E,F). As shown in Figure 5G‐I, DOX reduced the expression levels of *eNOS*, *sGC* and *PKG1* genes to values found in heart tissue in rats (*P* < .01), and the combination of HG and [6]‐GR ameliorated this decrease (*P* < .01). Overall, HG/[6]‐GR combination has a more significant effect than the two drugs used alone, which indicated the combination superiority of HG and [6]‐GR.

### 3.10 HG/[6]‐GR inhibits the expressions of ACE, AGTR1 and NRG1 in myocardial tissue of rats

The expressions of ACE, AGTR1 and NRG1 in cardiomyocytes were determined by immunohistochemical staining. The results showed that ACE, AGTR1 and NRG1 were significantly overexpressed in the DOX group compared with the control group (Figure 5J). The analysis showed that the expressions of ACE, AGTR1 and NRG1 in the HG and [6]‐GR groups were lower than the DOX group. HG/[6]‐GR obviously down‐regulated the expressions of ACE, AGTR1 and NRG1 versus the DOX group (Figure 5J).

### 3.11 HG/[6]‐GR combination up‐regulates relative expression of LKB1/AMPKα/Sirt1 pathway

**Figure 6:**
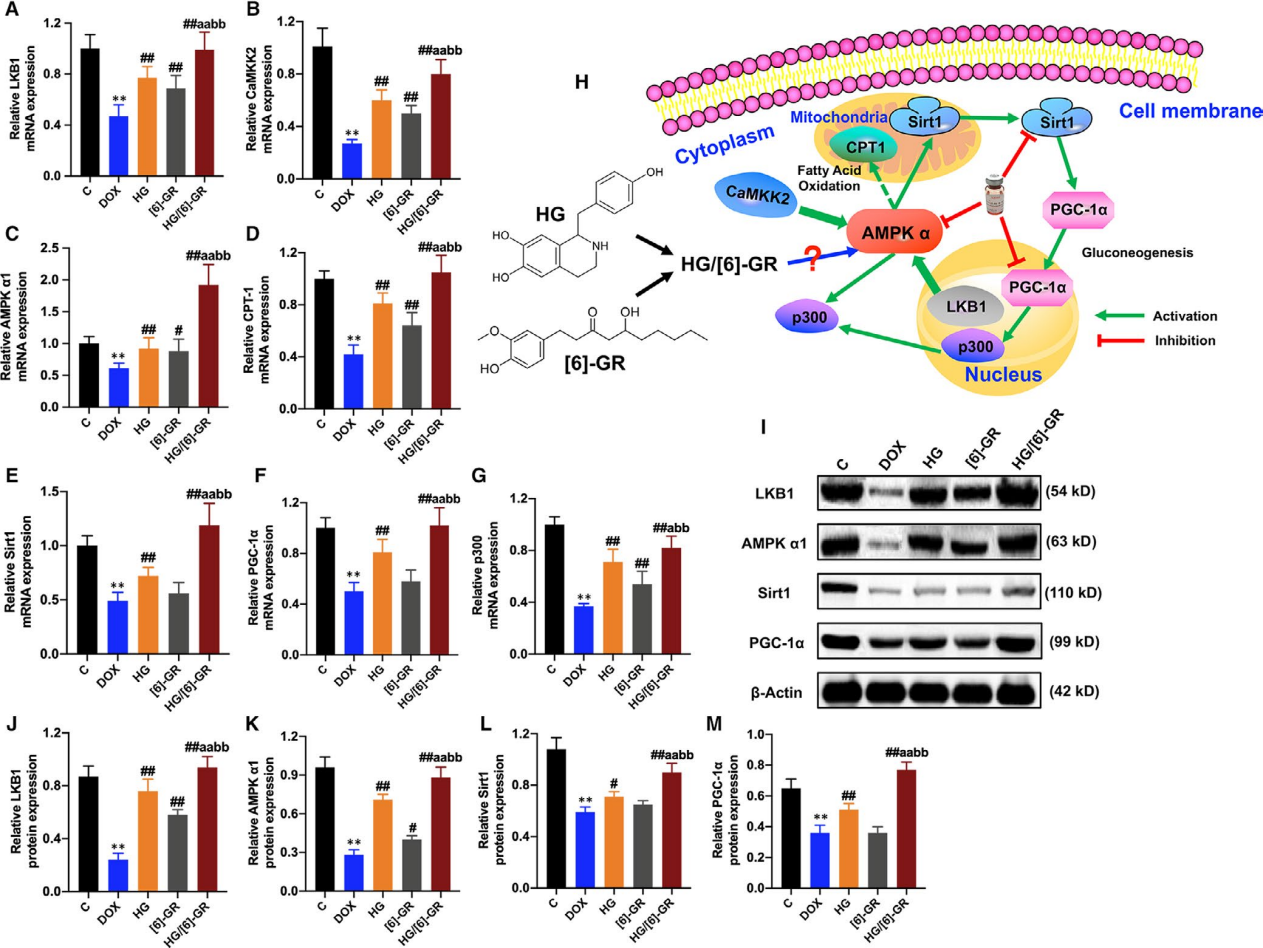
Effects of HG/[6]‐GR couple on the relative mRNA and protein expression levels in the heart tissue of rats. (A) Relative LKB1 mRNA level in heart tissue; (B) relative CaMKK2 mRNA level in heart tissue; (C) relative AMPK α1 mRNA level in heart tissue; (D) relative CPT‐1 mRNA level in heart tissue; (E) relative Sirt1 mRNA level in heart tissue; (F) relative PGC‐1α mRNA level in heart tissue; (G) relative p300 mRNA level in heart tissue; (H) schematic for the role of HG/[6]‐GR in LKB1/AMPK α1/Sirt1 signalling pathway in rats; (I) Western blotting images of LKB1, AMPK α1, Sirt1 and PGC‐1α; (J) relative LKB1 protein level in heart tissue; (K) relative AMPK α1 protein level in heart tissue; (L) relative Sirt1 protein level in heart tissue; (M) relative PGC‐1α protein level in heart tissue. ∗∗*P* < .01 compared with the control group; #*P* < .05, ##*P* < .01 compared with the DOX group; a*P* < .05, aa*P* < .01 compared with the HG group; b*P* < .05, bb*P* < .01 compared with the [6]‐GR group

As activated AMPK could monitor mitochondrial energy metabolism and cellular energy status,^27^ the relative mRNA and protein expression of upstream and downstream of the AMPK signalling pathway were detected. Thus, the relative mRNA and protein expression levels of the LKB1/AMPKα/Sirt1 pathway were measured by RT‐PCR and Western blotting, respectively (Figure 6). The results showed that the relative mRNA and protein expressions of LKB1, AMPK α1, Sirt1 and PGC‐1α in the DOX group decreased compared with the control group (*P* < .01). HG notably up‐regulated the expressions of these mRNA and proteins, and the expressions of them were further increased in the HG/[6]‐GR couple group versus the HG and [6]‐GR groups (*P* < .01; *P* < .05). The HG and [6]‐GR groups promoted these mRNA and protein levels in varying degrees (Figure 6).

## 4 DISCUSSION

Pathological mechanism and influencing factors of the occurrence and development of CHF are complex, involving haemodynamics, neurohormones, genetic factors, energy and lipid metabolism, etc. Professor Neubauer believes that the myocardial energy supply is insufficient or metabolic imbalance during the occurrence of CHF, which leads to the damage of cardiac structure and function thus causes HF.^28^ The exhausted heart is referred as “fuelled engines.” Professor Van Bilsen^29^ proposed the concept of "metabolic remodelling" in view of the occurrence and development of CHF. He believed that the abnormal changes in cardiac structure and function were caused by cardiac metabolic disorders. It is evident that exploring the metabolic pathways of CHF and improving cardiac metabolic disorders become one of the new effective ways to treat CHF. New ways of prevention and treatment of HF are of great benefit.

The neuroendocrine mechanism of CHF is the basis of classical drugs. After obtaining strong evidence to improve the prognosis of patients with HF through carefully designed randomized trials, drugs for the sympathetic nervous system and RAAS have become the pillars of CHF treatment.^30^ Therefore, a systematic study of drugs affecting RAAS may be of great significance for the treatment and prognosis of CHF. Previous studies have found that HG/[6]‐GR can promote mitochondrial energy metabolism and protects H9c2 cells from HF via up‐regulating the PPARα/PGC‐1α/Sirt3 pathways.^24^ However, the anti‐HF effect of HG/[6]‐GR has not been systematically studied in vivo. The results indicate that HG/[6]‐GR has effects on improving cardiac function, inhibiting cardiomyocyte apoptosis and affecting the RAAS system to play an anti‐CHF role in vivo. In addition, cell metabolomics coupled with Seahorse XF technology was performed to systematically predict and clarify the protective effects and potential mechanisms of HG/[6]‐GR in resisting DOX‐induced cardiac myocyte injury from the perspective of its influence on mitochondrial energy metabolism. It is helpful to find new targets and new drugs for the intervention of HF.

Advances in cell metabolomics have improved our understanding of HG/[6]‐GR for CHF treatment in several crucial ways. Firstly, cardiomyocyte metabolomic characteristics better define the pathogenesis of CHF. Secondly, the characteristics of potential biomarkers of cardiomyocyte metabolism are conducive to the discovery of candidate targets.^31^ Thirdly, changes in metabolic markers can be used to evaluate the effectiveness of drugs and its pharmacological interventions.^32^ Fourthly, the discovery of metabolic pathways can be used to analyse the potential mechanism of HG/[6]‐GR in the treatment of CHF. Most metabolites decreased in treatment with DOX. It is worth noting that elevation for some metabolites in the HG and [6]‐GR groups was relatively lower in comparison with the HG/[6]‐GR group, indicating that the combined use of HG and [6]‐GR has contributed to this inconsistency. The principal disturbed metabolic pathways based on the 8 differential metabolites include altered biosynthesis of unsaturated fatty acids, fatty acid metabolism, fatty acid biosynthesis and fatty acid elongation in mitochondria, as well as affected citrate cycle (TCA cycle). All these phenomena indicated that HG/[6]‐GR could alleviate DOX‐caused cardiomyocyte injury by regulating fatty acid metabolism and mitochondrial energy metabolism in the TCA cycle of H9c2 cardiomyocyte.

Agilent Seahorse XF technology can simultaneously measure the two main energy production pathways of cell mitochondrial respiration and glycolysis, which accelerates our understanding of cellular function, activation, proliferation, differentiation and disease aetiology. Combining cell energy, phenotype test with the cell extracellular flux analyser can quickly measure mitochondrial respiration and fuel usage. Therefore, the metabolic phenotypes and metabolic potential of H9c2 cells were measured in the current study. In addition, a cell mitochondrial fuel flex test was used to determine the oxidation rate of fatty acid by measuring mitochondrial respiration of H9c2 cells in the presence or absence of fatty acid pathway inhibitors. Of note, the combination of HG/[6]‐GR strongly ameliorated mitochondrial energy metabolism in terms of baseline OCR, baseline ECAR, stressed OCR and stressed ECAR. Moreover, these results indicated that fatty acid mitochondrial fuel usage of H9c2 cells was decreased as a result of DOX treatment compared to the control group. However, OCR, dependency, capacity and flexibility of fatty acids were increased in the HG and [6]‐GR combination groups compared to the DOX group.

As mitochondrial dysfunction plays a crucial role in energy homoeostasis, metabolism and signalling,^33^ and activating the LKB1/AMPK/Sirt1 pathway prevents the development of cardiomyopathy by improving lipid and fatty acid metabolism,^34^ mitochondrial energy metabolism‐related AMPK/Sirt1 pathway was investigated to evaluate whether HG/[6]‐GR had potential effect on LKB1/AMPK/Sirt1 axis and its downstream targets. Researches have shown that LKB1/AMPK/Sirt1 axis is involved in a variety of biological processes during cell growth.^35^ It is noteworthy that the LKB1/AMPK/Sirt1 axis and its downstream targets are highly sensitive targets for DOX‐induced cardiac injury.^36^ SIRT1 is reported to have a critical role in controlling the cardiac LKB1/AMPK pathway.^37^ Activated Sirt1 can also regulate the activity of the peroxisome proliferator‐activated receptor gamma coactivator 1 alpha (PGC‐1α) and further up‐regulate its expression.^38^ These signalling pathways are energy‐sensing networks and play an important regulatory role in mitochondrial biosynthesis and energy metabolism.^39^ In the current study, the results showed that HG/[6]‐GR promotes the mRNA and protein expression of LKB1, AMPK α1, SIRT1 and PGC‐1α. Then, it can prevent the heart from DOX‐induced mitochondrial function disorder.

Although integrated approaches were used to clarify the effectiveness and mechanism of HG combined with [6]‐GR in the treatment of CHF, there still has some limitations: (a) this study explored the therapeutic effect and potential mechanism of action of HG combined with [6]‐GR on DOX‐induced CHF model. However, the cardiotonic and anti‐CHF effects of other active compounds in ALRP and ZR, such as coryneine chloride, fuzinoside and uracil, have not been explored yet. (b) The role of HG and [6]‐GR on other types of CHF models besides DOX remains to be clarified. (c) The therapeutic effects of HG/[6]‐GR in other types of CAD, such as myocardial ischaemia, arrhythmia and coronary heart disease, need to be further explored. (d) Although the effects of HG/[6]‐GR on several mitochondrial biomarkers of cardiomyocytes have been studied at the cellular level, the effects of HG/[6]‐GR on the glycolytic rate, glycolysis stress, glycolytic ATP production rate, mitochondrial ATP production rate, total ATP production rate, proton efflux rate and other parameters of HG/[6]‐GR‐treated H9c2 cells need further study.

## 5 CONCLUSION

In conclusion, this study demonstrated that HG and [6]‐GR may be the active component of ALRP, ZR, respectively, which can therapy DOX‐induced CHF and ameliorate H9c2 cells from DOX‐induced cardiomyocyte toxicity. The molecular mechanism responsible for cardiomyocyte activity of HG/[6]‐GR may involve the promoting mitochondrial energy metabolism signalling pathway, which alleviates DOX‐induced mitochondrial respiratory function impairment and energy metabolism disorders to improve H9c2 cells' dysfunction. This study will provide the theoretical fundament for the further acquaintance of the compatibility theory of Chinese material medical and have a critical significance for the guidance of the clinic practice of TCM.

## CONFLICT OF INTEREST

The authors declare that there are no conflicts of interest.

## AUTHOR CONTRIBUTIONS

Wenjun Zou and Yanling Zhao conceived and designed the study. Jianxia Wen and Lu Zhang performed the experiments and wrote the manuscript. Jian Wang and Jiabo Wang collected and prepared samples. Lifu Wang, Ruilin Wang and Ruisheng Li analysed the data. Honghong Liu, Shizhang Wei and Haotian Li amended the manuscript. All authors read and approved the final version of the manuscript.

## ETHICAL APPROVAL

All animal procedures complied with the Guiding Principles for the Care and Use of Laboratory Animals of China and Institutional Animal Care and Use Committee of the Fifth Medical Center of PLA General Hospital. All animal studies were approved by the Committee on the Ethics of Animal Experiments of the Fifth Medical Center of PLA General Hospital (Approval ID: IACUC‐2018‐010).

## Data Availability

The data used to support the findings of this study are available from the corresponding author upon reasonable request.
