# Supplementary figures and images for: Therapeutic effects of higenamine combined with [6]‐gingerol on chronic heart failure induced by doxorubicin via ameliorating mitochondrial function
Source: J Cell Mol Med. 2020 Feb 19;24(7):4036–50. doi: 10.1111/jcmm.15041 (PMC7171398; doi:10.1111/jcmm.15041)

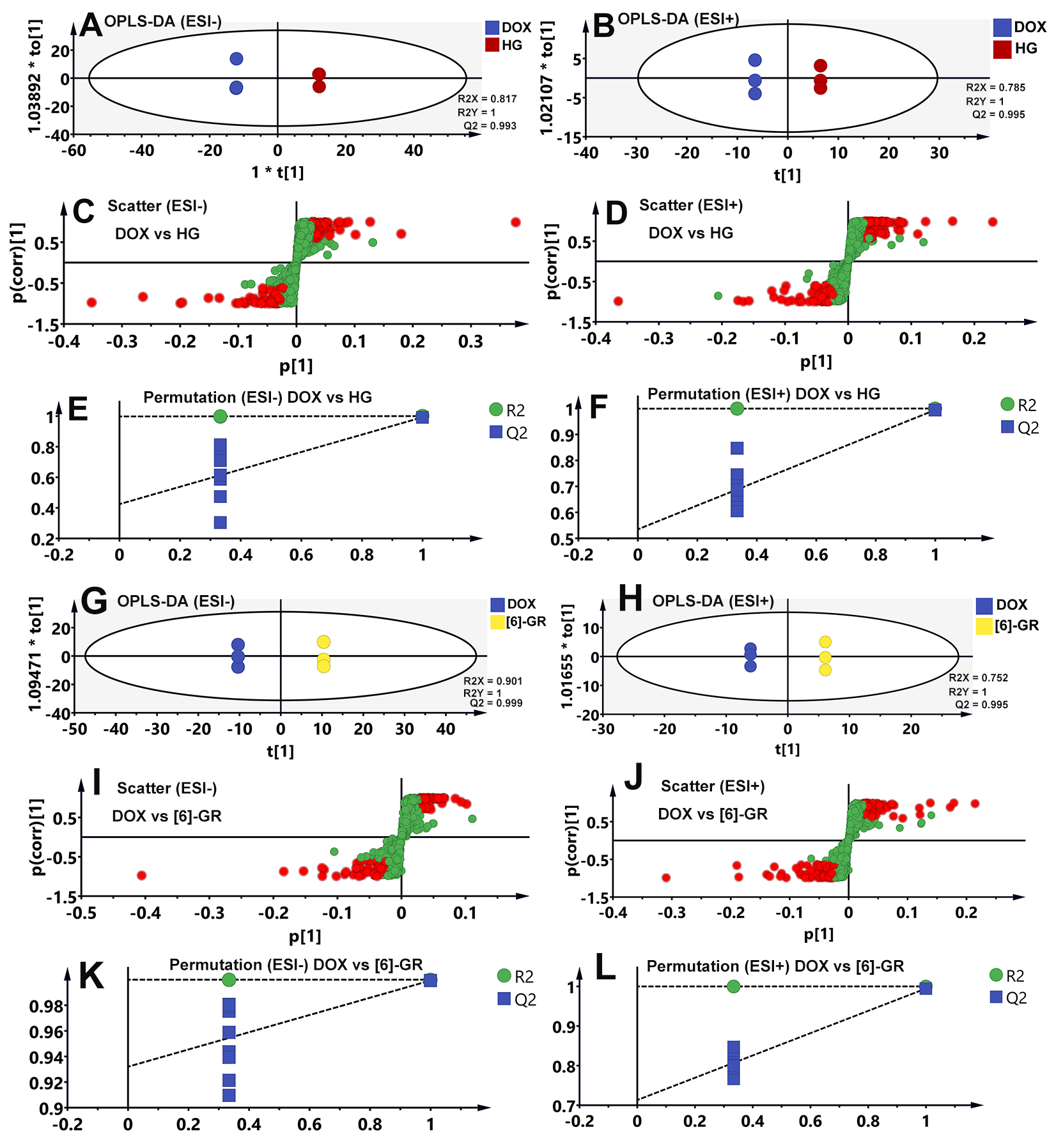

Supplement: Supplementary file 1 [file JCMM-24-4036-s001.tif]
