# Supplementary material for: Therapeutic effects of higenamine combined with [6]‐gingerol on chronic heart failure induced by doxorubicin via ameliorating mitochondrial function
Source: J Cell Mol Med. 2020 Feb 19;24(7):4036–50. doi: 10.1111/jcmm.15041 (PMC7171398; doi:10.1111/jcmm.15041)
Supplement: Supplementary file 2 [file JCMM-24-4036-s002.docx]

**TABLE S1** Primers sequences for RT-PCR analyses in this study.

| Gene | Forward | Reverse | prodSize |
| --- | --- | --- | --- |
| *PKG1* | GCTGCCTTCTTCGCCAACCTG | TGCTCCTGCTGTCTCGTGTCC | 170 |
| *ACE* | GACGGAAGCATCACCAAGGAGAAC | CATAGTGCCTCGTGGAACTGGAAC | 197 |
| *AT1R* | GCTTCAACCTCTACGCCAGTGTG | CAGCCAGATGATGATGCAGGTGAC | 137 |
| *cGRP* | AACAGGAGGAGCAAGAGACTGAGG | GGTGGCGGTGTTGCAGGATC | 86 |
| *eNOS* | CACAGGCATCACCAGGAAGAAGAC | TTCACACGCTTCGCCATCACC | 96 |
| *cGKI* | GCTGCCTTCTTCGCCAACCTG | TGCTCCTGCTGTCTCGTGTCC | 170 |
| *NRG1* | GCTGGTGGTCGGCATCATGTG | GTTGCTCCGTTCTGACCGAAGAC | 106 |
| *AngPTL4* | CATGGCTGCCTGCGGTAACG | TCTGGAACAGTTGCTGGATCTTGC | 146 |
| *sGC* | TTCTCCTTACTGGCTCCTGGTCAG | GTAGGTAGACTCTGTTGCGGCTTG | 163 |
| *FAS* | GTCCTGCCTCTGGTGCTTGC | TTCACGAACGCTCCTCTTCAACTC | 99 |
| *CPT-1* | CAACATCCTGTCCACCAGCACTC | GCAGCCTATCCAGTCATCGTGAAC | 118 |
| *AMPK α1* | TTGCGTGTGCGAAGGAAGAACC | CCGATCTCTGTGGAGTAGCAGTCC | 157 |
| *Sirt1* | GCTCGCCTTGCTGTGGACTTC | GTGACACAGAGATGGCTGGAACTG | 141 |
| *PGC-1α* | GATGGAGACGTGACCACTGACAAC | GTGCTAAGACCGCTGCATTCATTG | 158 |
| *P300* | AACAACCAGCAGCAACAGCAATTC | CAGCAGGAGGAACAGGAAGTGAAG | 162 |
| *CaMKK2* | CAGCCTGACATAGCCGAAGACTTG | CACCTCGACCAGTGTGCAGTTC | 165 |
| *LKB1* | AGAGGAGGAGGAGGACGAGGAC | TGGGCAGGCTGTGGCTCTG | 128 |
| *β-Actin* | CACTATCGGCAATGAGCGGTTCC | CAGCACTGTGTTGGCATAGAGGTC | 154 |
