# Supplementary material for: Therapeutic effects of higenamine combined with [6]‐gingerol on chronic heart failure induced by doxorubicin via ameliorating mitochondrial function
Source: J Cell Mol Med. 2020 Feb 19;24(7):4036–50. doi: 10.1111/jcmm.15041 (PMC7171398; doi:10.1111/jcmm.15041)
Supplement: Supplementary file 3 [file JCMM-24-4036-s003.docx]

**TABLE S2** Changes of myocardial hemodynamic parameters in rats.

| Group | n | LVSP (mmHg) | LVEDP (mmHg) | ＋dp/dt_max_ (mmHg/s) | -dp/dt_max_ (mmHg/s) |
| --- | --- | --- | --- | --- | --- |
| C | 3 | 129.23 ± 10.55 | -0.37 ± 0.14 | 7683.02 ± 419.88 | -5377.57 ± 466.58 |
| DOX | 3 | 53.53 ± 8.07^**^ | 11.95 ± 1.27^**^ | 2580.73 ± 504.42^**^ | -1764.38 ± 302.05^**^ |

All data were presented as the mean ± SD. (n=3). ^**^*P*<0.01, compared with the control group.
